# Glucose-induced gradual phenotypic modulation of cultured human glomerular epithelial cells may be independent of Wilms’ tumor 1 (WT1)

**DOI:** 10.1186/1471-2121-14-28

**Published:** 2013-06-14

**Authors:** Nikolaos E Tsotakos, Marina Sagnou, Eleni S Kotsopoulou, Effie C Tsilibary, Garyfalia I Drossopoulou

**Affiliations:** 1 Institute of Biosciences and Applications, National Centre for Scientific Research “Demokritos”, Athens, Greece

**Keywords:** Podocalyxin, Nephrin, Dedifferentiation, Podocytes, WT1

## Abstract

**Background:**

Renal podocytes form the main filtration barrier possessing a unique phenotype maintained by proteins including podocalyxin and nephrin, the expression of which is suppressed in pathological conditions. We used an *in vitro* model of human glomerular epithelial cells (HGEC) to investigate the role of high glucose in dysregulating the podocytic epithelial phenotype and determined the time needed for this change to occur.

**Results:**

In our *in vitro* podocyte system changes indicating podocyte dedifferentiation in the prolonged presence of high glucose included loss of podocalyxin, nephrin and CD10/CALLA concomitant with upregulation of mesenchymal vimentin. Our study demonstrates for the first time that podocyte-specific markers undergo changes of expression at different time intervals, since glucose-mediated podocalyxin downregulation is a progressive process that precedes downregulation of nephrin expression. Finally we demonstrate that high glucose permanently impaired WT1 binding to the podocalyxin gene promoter region but did not affect WT1 binding on the nephrin gene promoter region.

**Conclusion:**

The presence of high glucose induced a phenotypic conversion of podocytes resembling partial dedifferentiation. Our study demonstrates that dysregulation of the normal podocytic phenotype is an event differentially affecting the expression of function-specific podocytic markers, exhibiting downregulation of the epithelial marker CD10/CALLA and PC first, followed by stably downregulated nephrin. Furthermore, it is herein suggested that WT1 may not be directly involved with upregulation of previously reduced PC and nephrin expression.

## Background

Glomerular visceral epithelial cells or podocytes comprise the outermost layer of the glomerular filtration apparatus. The podocytic slit diaphragm (SD) is a specialized cell junction that keeps adjacent foot processes inter-connected [1,2]. The loss of podocyte-specific proteins is reminiscent of dedifferentiation, which has also been described as epithelial-to-mesenchymal transition (EMT) [3]. Tubular epithelial cells also undergo dedifferentiation *in vitro* after incubation with fibrogenic TGF-β [4]. Injurious stimuli trigger different responses, ranging from podocytic hypertrophy and detachment to apoptosis [3]. Under these pathological conditions podocytes lose their specialized features and phenotype and may acquire mesenchymal markers [3,5]. This has been shown to be the case in HIV-induced nephropathy and collapsing glomerulopathy [6] as well as TGF-β-induced podocyte injury [7].

Pivotal podocytic markers include the antiadhesive protein podocalyxin (PC), which regulates podocyte morphology, as well as foot process formation and maintenance [8,9] and the SD-specific transmembrane protein nephrin, which is also implicated in the pathophysiology of proteinuria [10]. Glucose induces PC suppression *in vivo*, in glomeruli of streptozotocin-diabetic rats [6] and *in vitro* in human glomerular epithelial cells (HGEC) [11,12]. Nephrin reduction in HGEC can be induced by glucose [12] and may be related to side-effects of glycated albumin/AGEs [13]. The intracellular domain of nephrin associates with CD2AP [14], an adaptor molecule which plays a major role in the maintenance of podocyte phenotype due to its cytoskeleton stabilizing properties [15]. A cell surface marker that has long been considered a differentiation marker of renal epithelium is Common Acute Lymphoblastic Leukemia Antigen (CALLA, also called CD10) [16,17]. Another protein used as a differentiation marker is vimentin, an intermediate filament protein characteristic of cells of mesenchymal origin. Upregulation of its expression is considered a major criterion for EMT [18-20] and of podocyte injury as reported in PAN nephrosis in rats [21].

HGEC exhibit a regular cobblestone appearance in culture and their phenotype agrees with that of parental podocytes [11,17]. Glucose-induced PC supression in HGEC cannot be restored by reverting glucose concentration to normal levels for either short or longer time intervals [12]. Therefore, we investigated whether HGEC exposure to high glucose resulted in loss of the differentiated podocytic characteristics and determined the time points when this phenotypic modulation takes place. Our results indicated that loss of PC surface expression coincided with reduced CD10/CALLA surface levels, while CD2AP expression was not altered. Moreover, loss of nephrin expression accompanied the glucose-induced downregulation of PC and CD10/CALLA, establishing that suppression of PC surface expression occurred earlier, when other pivotal podocytic markers were still unaffected. These observations indicated that PC downregulation occurs in podocytes still possessing some of their characteristics.

## Results

### Transient culture of HGEC in high glucose resulted in reversible upregulation of vimentin protein expression

**Figure 1:**
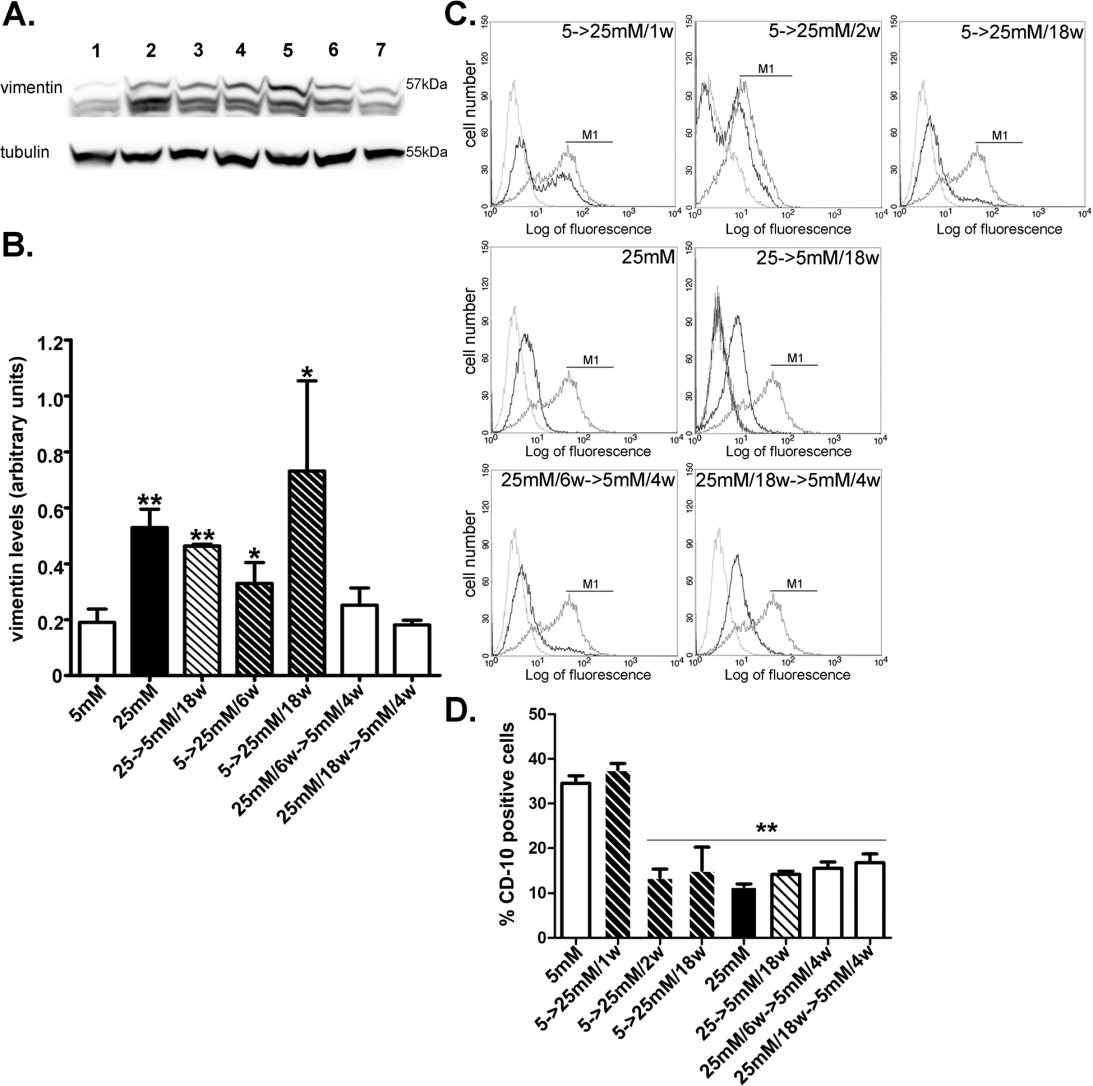
**Vimentin and CD10/CALLA expression in podocytes.** (**A**) Western blot analysis for vimentin expression in HGEC. Total protein was extracted from cells cultured in the presence of 5 mM (control) (lane 1) or 25 mM (lane 2) glucose. Lane 3 represents vimentin expression in HGEC continuously exposed to 25 mM and reverted to 5 mM glucose for 18 weeks (HGEC:25 mM-to-5 mM/18w). Lanes 4 and 5 represent HGEC continuously exposed to 5 mM glucose and reverted to 25 mM for 6 and 18 weeks respectively (HGEC:5 mM-to-25 mM/6w, HGEC:5 mM-to-25 mM/18w). Lanes 6 and 7 represent HGEC:25 mM/6w-to-5 mM/4w and HGEC:25 mM/18w-to-5 mM/4w respectively. For quantification of vimentin the 57 kDa band was used. Blots were reprobed with anti-tubulin antibody, to verify protein loads, against which the data were normalized. (**B**) Results were expressed as the mean ± SD of three independent experiments. (**C**) Representative experiments from flow cytometric analysis of CD10/CALLA. Thick grey lines represent HGEC:5 mM, thin grey lines represent the corresponding isotype control antibody and black lines represent stimuli. (**D**) Percentage of positive cells was calculated for the histogram section indicated as M1 (**p<0.01 vs. HGEC:5 mM).

Vimentin is a well-known mesenchymal marker and its upregulation is considered a significant marker of dedifferentiation [19] and podocyte injury [22]. Since HGEC permanently grown in 25 mM glucose (HGEC:25 mM) display almost totally suppressed PC levels, compared to HGEC exposed to 5 mM glucose (HGEC:5 mM) [12], we examined whether this change could be attributed to dysregulation of the podocytic phenotype, earmarked by enhanced vimentin expression. Western blot analysis demonstrated that vimentin expression was upregulated in HGEC:25 mM (Figure 1A-B). Elevated vimentin expression levels were established following 6 weeks of culture in 25 mM glucose (HGEC:5 mM-to-25 mM/6w) (Figure 1A-B). Vimentin expression reached maximal levels following 18 weeks of culture (with serial passages) in 25 mM glucose (HGEC:5 mM-to-25 mM/18w) (Figure 1A-B), suggesting that modulation of the podocytic characteristics occurred progressively with time. In order to determine the time points at which alterations in expression levels of vimentin, as well as other important proteins expressed in podocytes occurred, HGEC were exposed to 25 mM glucose for 1, 2, 4, 6, 18 weeks. From this time course an early time point (6 weeks) and a late time point (18 weeks) were selected in order to investigate whether glucose effects were reversible. The early time point was chosen because it signifies upregulation of the mesenchymal marker vimentin and the late time point was chosen because alterations were maximal. Accordingly, we then examined whether the observed change in vimentin expression could be restored to normal levels, at the early (6 weeks) time point (HGEC:5 mM-to-25 mM/6w) and the late (18 weeks) time point (HGEC:5 mM-to-25 mM/18w). HGEC exposed to 25 mM glucose for 6 weeks were reverted to normal glucose levels (5 mM) for another 4 weeks (HGEC:25 mM/6w-to-5 mM/4w). Additionally HGEC:5 mM-to-25 mM/18w were cultured in 5 mM glucose for 4 more weeks (HGEC:25 mM/18w-to-5 mM/4w). In both time intervals, early and late, vimentin reverted to lower, normal levels of expression.

### *In vitro* culturing of HGEC in the presence of high glucose levels resulted in permanent downregulation of CD10/CALLA protein expression

We examined the expression of CD10/CALLA in a similar manner to vimentin. FACS analysis showed that HGEC:5 mM expressed CD10/CALLA (Figure 1C,D). On the contrary, HGEC:25 mM demonstrated significantly reduced cell surface-associated levels (Figure 1C,D). Significantly reduced CD10/CALLA surface levels were established following 2 weeks of culture in 25 mM glucose (HGEC:5 mM-to-25 mM/2w) (Figure 1C,D) and remained downregulated following 6 (data not shown) and 18 weeks of culture in 25 mM glucose (HGEC:5 mM-to-25 mM/6w, HGEC:5 mM-to-25 mM/18w) (Figure 1C,D). CD10/CALLA surface levels remained significantly reduced after reverting glucose concentration to 5 mM for 18 weeks (HGEC:25 mM-to-5 mM/18w) (Figure 1C,D). We next examined whether the observed downregulation of CD10/CALLA could be reversed at early (6 weeks) and late (18 weeks) time intervals, in HGEC sequentially grown in 5 mM glucose (HGEC:25 mM/6w-to-5 mM/4w and HGEC:25 mM/18w-to-5 mM/4w). In both instances CD10/CALLA remained reduced suggesting permanent glucose-induced downregulation of its expression.

### Reversible phenotypic changes of expression in cultured podocytes are accompanied by normal levels of cell surface-associated nephrin

HGEC:25 mM displayed severely reduced nephrin expression compared to HGEC:5 mM [11,12]. Nephrin expression remained suppressed even 12 weeks after reverting glucose concentration to 5 mM glucose levels [12].

**Figure 2:**
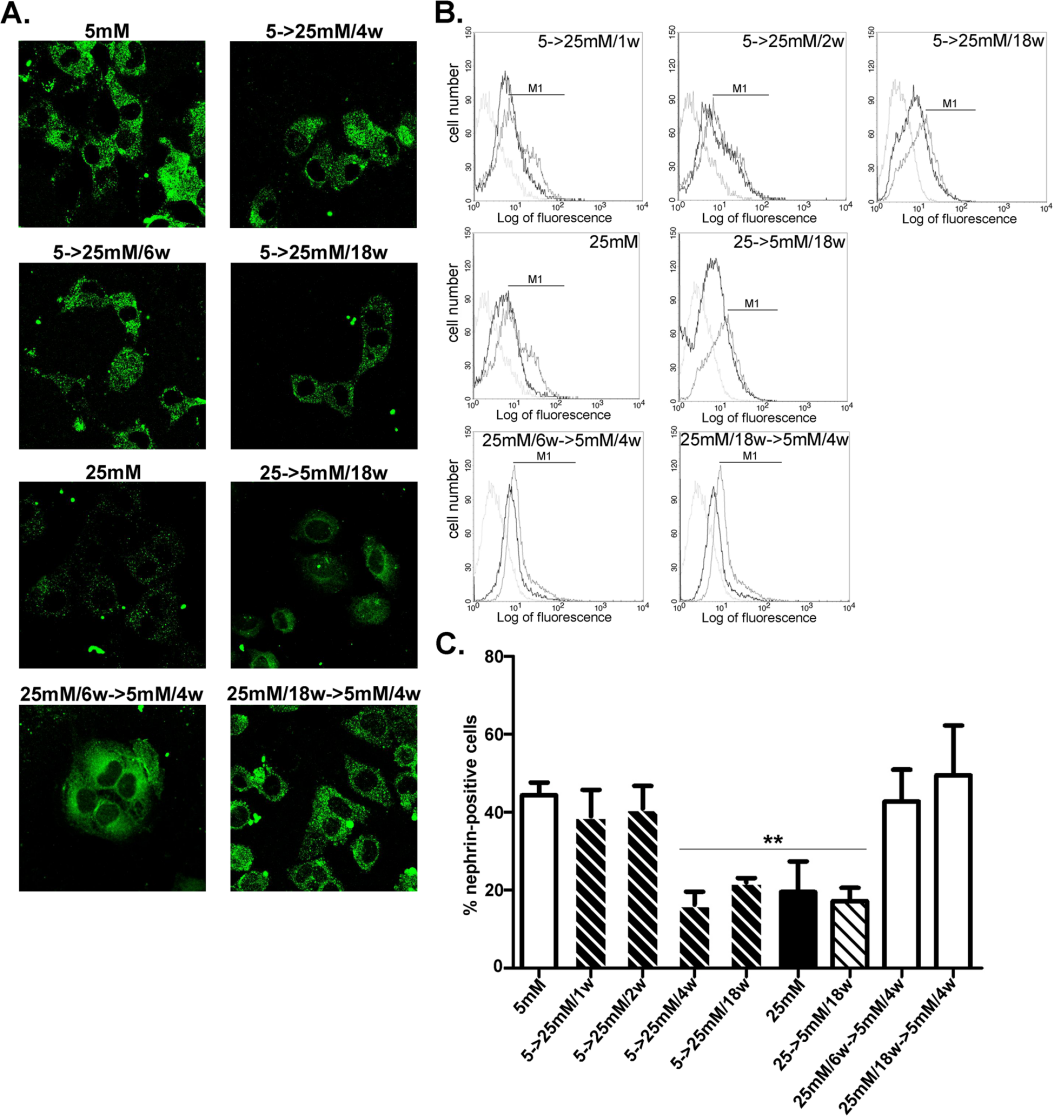
**Nephrin cell surface expression in podocytes.** (**A**) Confocal microscopic analysis (×600) for the distribution of nephrin in HGEC. Cells were fixed and stained with anti-nephrin antibody and AlexaFluor 488-conjugated anti-goat IgG as secondary antibody. (**B**) FACS analysis of nephrin cell surface expression. Representative experiments from flow cytometric analysis of nephrin. Thick grey lines represent HGEC 5 mM, thin grey lines represent the corresponding isotype control antibody and black lines represent stimuli. (**C**) Percentage of positive cells was calculated for the histogram section indicated as M1 (**p<0.01 vs. HGEC:5 mM).

**Figure 3:**
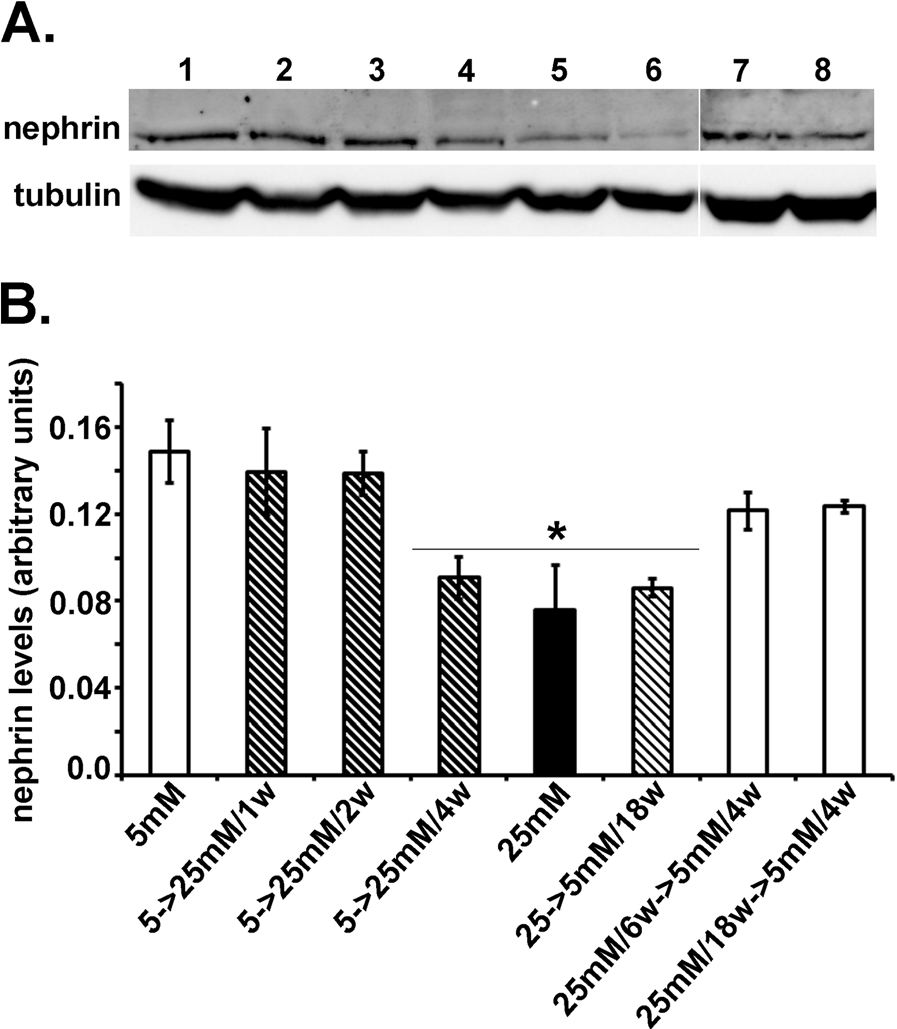
**Nephrin expression in podocytes.** (**A**) Western blot analysis for nephrin expression in HGEC. Total protein was extracted from cells cultured either in the presence of 5 mM (control) (lane 1) or 25 mM (lane 5) glucose. Lanes 2, 3 and 4 represent HGEC:5 mM-to-25 mM/1w, HGEC:5 mM-to-25 mM/2w and HGEC:5 mM-to-25 mM/4w respectively. Lane 6 represents HGEC:25 mM-to-5 mM/18w. Lanes 7 and 8 represent HGEC:25 mM/6w-to-5 mM/4w and HGEC:25 mM/18w-to-5 mM/4w respectively. Blots were reprobed with anti-tubulin antibody, to verify protein loads, against which the data were normalized. (**B**) Results were expressed as the mean ± SD of four independent experiments (*p<0.05 vs. HGEC:5 mM).

Since nephrin associated with cell surface was demonstrated to play a major role in the development of proteinuric diseases [23], we next examined cell surface-associated nephrin by immunocytochemical and FACS approaches. In HGEC:5 mM-to-25 mM/4w, surface nephrin levels were downregulated (Figure 2A,C) and remained reduced in HGEC:5 mM-to-25 mM/6w and HGEC:5 mM-to-25 mM/18w as well as in HGEC 25 mM-to-5 mM/18w (Figure 2). However, in HGEC:25 mM/6w-to-5 mM/4w, and HGEC:25 mM/18w-to-5 mM/4w_,_ cell surface-associated nephrin was restored to normal levels, similar to those observed in HGEC:5 mM (Figure 2). In order to verify whether these findings were not only associated with cell surface nephrin expression but may also correspond to total protein levels, immunoblotting experiments were performed that confirmed our immunocytochemical and FACS analyses (Figure 3).

### *In vitro* culturing of HGEC in high glucose transiently reduced CD2AP expression

**Figure 4:**
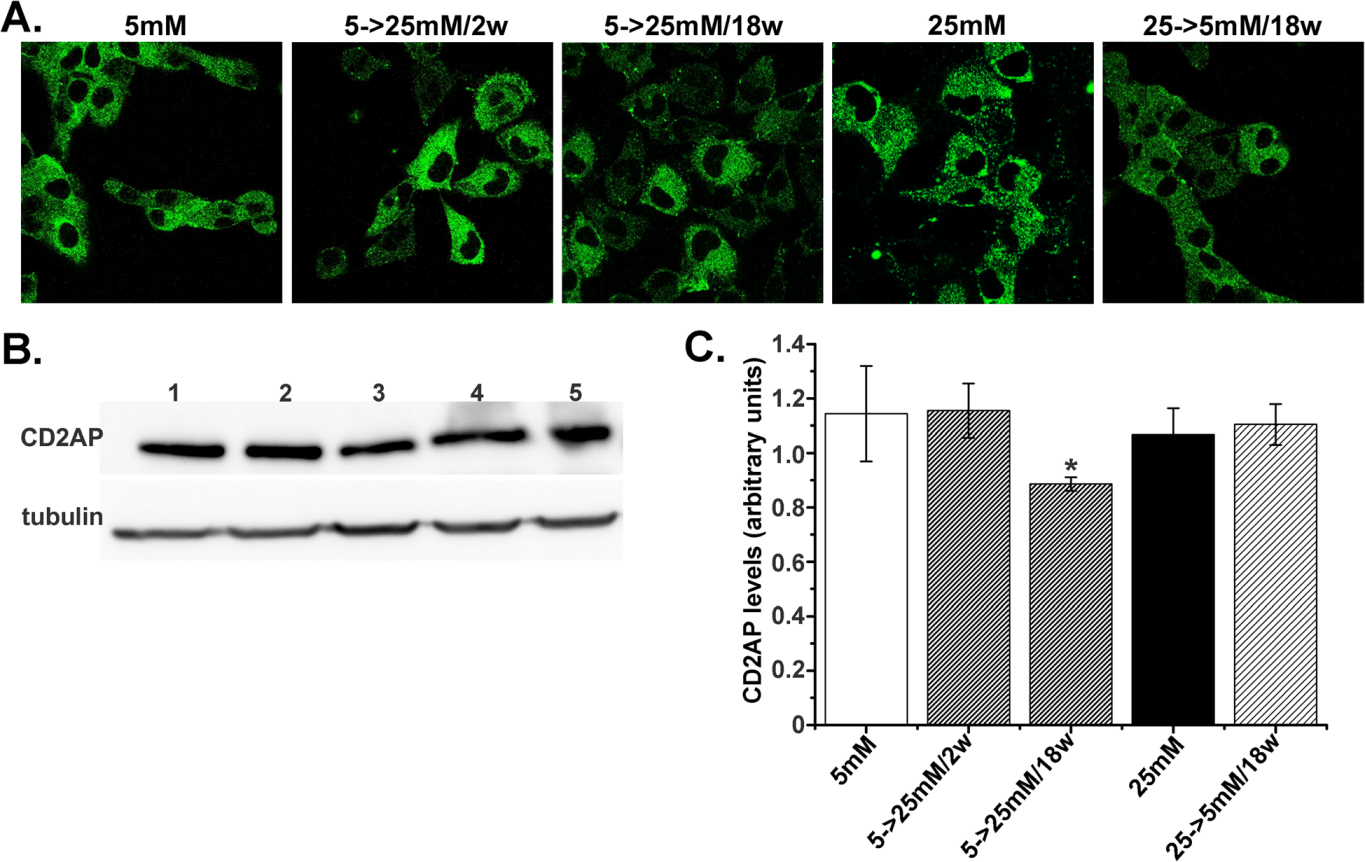
**CD2AP expression in podocytes.** (**A**) Confocal microscopic analysis (×600) for the distribution of CD2AP in HGEC. Cells were fixed and stained with anti-CD2AP antibody and AlexaFluor 488-conjugated anti-mouse IgG as secondary antibody. (**B**) Western blot analysis for CD2AP expression in HGEC. Total protein was extracted from HGEC:5 mM (control) (lane 1) or HGEC:25 mM (lane 4). Lanes 2 and 3 represent CD2AP expression in HGEC:5 mM-to-25 mM/2w and HGEC:5 mM-to-25 mM/18w respectively. Lane 5 represents CD2AP expression in HGEC:25 mM-to-5 mM/18w. Blots were reprobed with anti-tubulin antibody, to verify protein loads, against which the data were normalized. (**C**) Results were expressed as the mean ± SD of three independent experiments (*p<0.05 vs. HGEC:5 mM).

Western blot analysis and immunofluorescence studies indicated that cell surface expression of CD2AP was not affected by high glucose at 2 weeks (HGEC:5 mM-to-25 mM/2w) (Figure 4). HGEC:5 mM and HGEC:25 mM displayed a punctuate pattern of CD2AP expression (Figure 4A). Significantly reduced CD2AP expression was observed following 18 weeks of exposure to 25 mM glucose (HGEC:5 mM-to-25 mM/18w) (Figure 4B lane 4). However, CD2AP levels were not reduced in HGEC permanently grown in 25 mM glucose (HGEC:25 mM) (Figure 4). Although CD2AP expression was not affected, actin staining appeared reduced in HGEC:25 mM and actin fibers were dysregulated (Additional file 1: Figure S1). However, F-actin distribution could be readily restored after exposure of HGEC:25 mM to normal glucose levels for 96 hours (Additional file 1: Figure S1).

### Glucose-induced downregulation of PC expression is partly reversible

**Figure 5:**
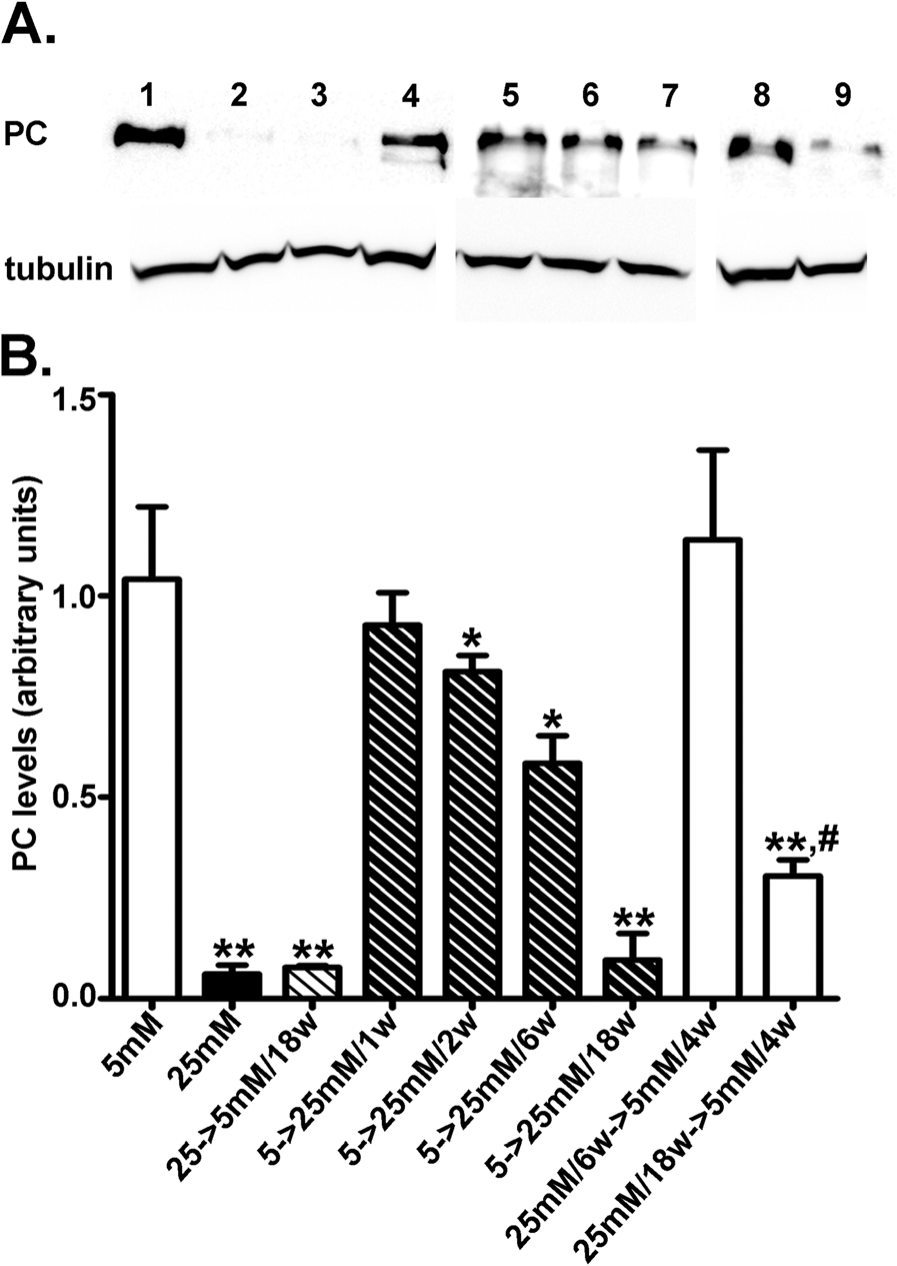
**PC expression in podocytes.** (**A**) Western blot analysis for PC expression in HGEC. Total protein was extracted from cells cultured either in the presence of 5 mM (control) (lane 1) or 25 mM (lane 2) glucose. Lane 3 represents PC expression in HGEC:25 mM-to-5 mM/18w. Lanes 4, 5, 6 and 7 represent HGEC:5 mM-to-25 mM/1w, HGEC:5 mM-to-25 mM/2w, HGEC:5 mM-to-25 mM/6w and HGEC:5 mM-to-25 mM/18w respectively. Lanes 8 and 9 represent HGEC:25 mM/6w-to-5 mM/4w and HGEC:25 mM/18w-to-5 mM/4w respectively. Blots were reprobed with anti-tubulin antibody, to verify protein loads, against which the data were normalized. (**B**) Results were expressed as the mean ± SD of four independent experiments (*p<0.05 vs. HGEC:5 mM, **p<0.01 vs. HGEC:5 mM, #p<0.05 vs. HGEC:25 mM).

**Figure 6:**
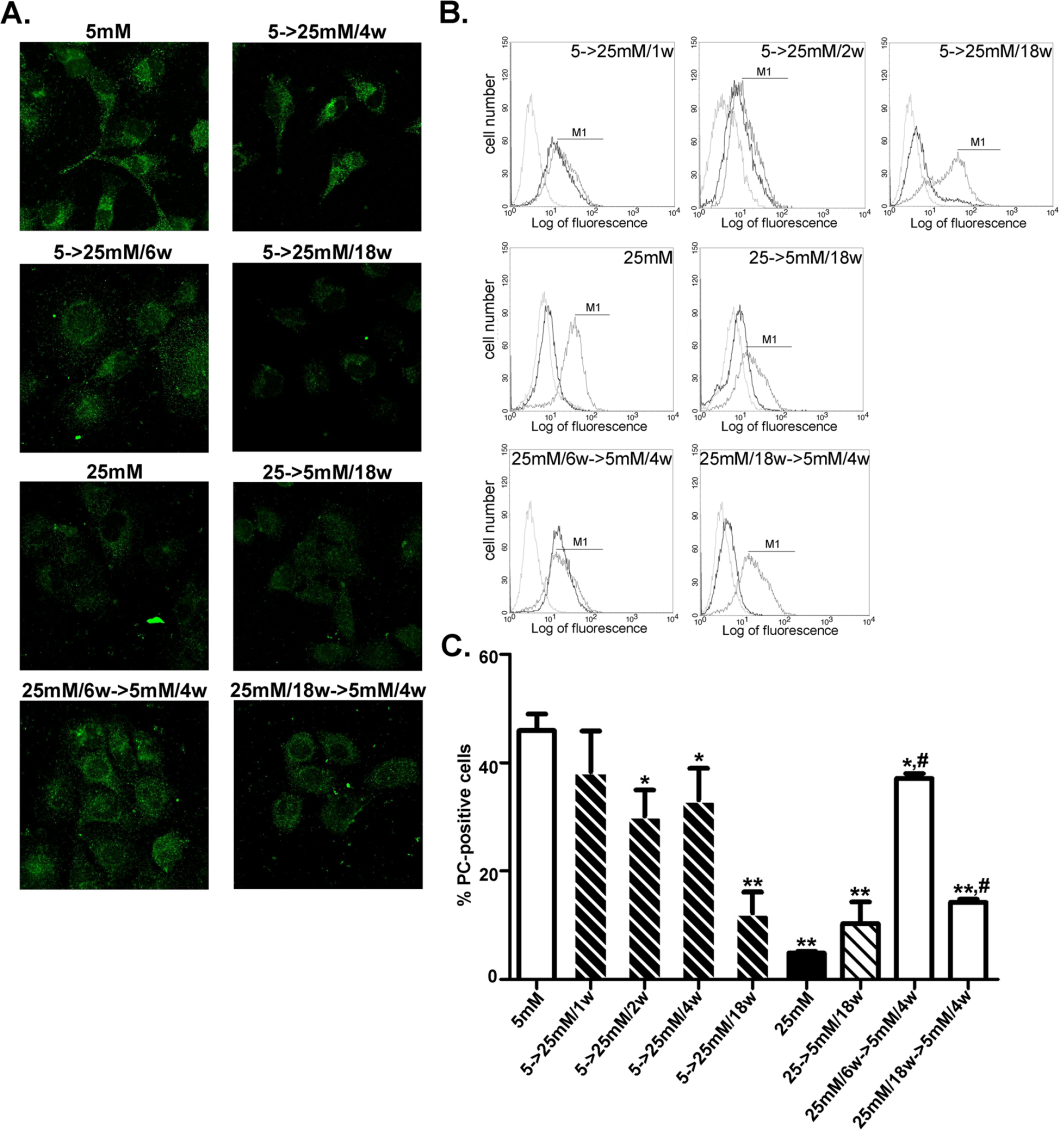
**PC cell surface expression in podocytes.** (**A**) Confocal microscopic analysis (×600) for the distribution of PC in HGEC. Cells were fixed and stained with anti-PC antibody and AlexaFluor 488-conjugated anti-mouse IgG as secondary antibody. (**B**) FACS analysis of PC cell surface expression. Representative experiments from flow cytometric analysis of PC. Thick grey lines represent cells HGEC:5 mM, thin grey lines represent the corresponding isotype control antibody and black lines represent stimuli. (**C**) Percentage of positive cells was calculated for the histogram section indicated as M1 (*p<0.05 vs. HGEC:5 mM, **p<0.01 vs. HGEC:5 mM, #p<0.05 vs. HGEC:25 mM).

Since HGEC:25 mM exhibit permanent PC expression downregulation [12], we performed a time-course using Western blot analysis to unravel when this phenotypic modulation occurs. Immunoblotting experiments showed that glucose-induced reduction of PC expression started at 2 weeks of culture in 25 mM glucose (HGEC:5 mM-to-25 mM/2w) and maximal downregulation was observed after 18 weeks of culture in 25 mM glucose (HGEC:5 mM-to-25 mM/18w) (Figure 5 lanes 5,7). The extent of PC downregulation in HGEC:5 mM-to-25 mM/2w and HGEC:5 mM-to-25 mM/6w was statistically significant, but PC expression levels remained higher than those observed in HGEC:25 mM (Figure 5B lanes 1, 2, 5, 6). In HGEC:5 mM-to-25 mM/18w, PC expression became maximally suppressed (Figure 5B lanes 2, 7). Similar findings were obtained for cell-surface PC levels, as assessed by immunocytochemical and FACS analyses (Figure 6). In HGEC exposed to 25 mM glucose for 6 weeks (early time interval) and then reverted to 5 mM glucose for four more weeks (HGEC:25 mM/6w-to-5 mM/4w), PC expression was totally restored (Figure 5A,B). On the contrary, HGEC cultured in 25 mM glucose for 18 weeks (late time interval) and reverted to normal glucose (5 mM) for four more weeks (HGEC:25 mM/18w-to-5 mM/4w) did not upregulate PC expression (Figure 5A,B). These observations were confirmed by FACS and immunofluorescence analyses (Figure 6). The observed effects were specifically due to D-glucose and not to any osmotic effect, because immunoblotting analysis of cells grown in 25 mM L-glucose revealed no changes in protein levels. The induced suppression was specific to PC, since other inter-related proteins such as α3β1-integrins were also decreased by high glucose, but their levels were immediately restored when glucose levels were reverted to 5 mM (Additional file 2: Figure S2).

### High glucose impaired binding of WT1 to the PC promoter

**Figure 7:**
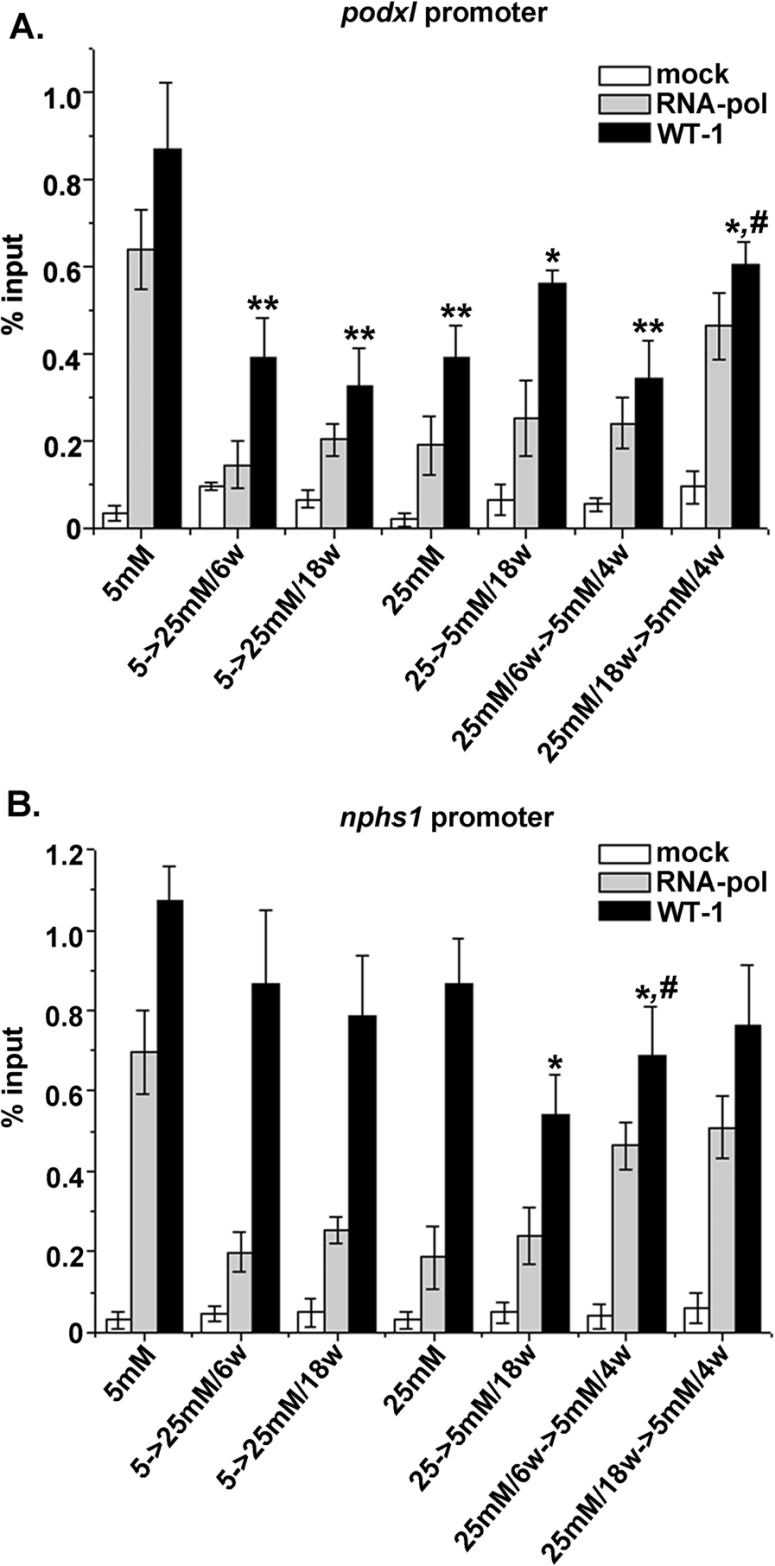
**ChIP analysis of WT1 binding to *podxl *and *nphs1 *promoter regions.** (**A**) WT1 binds *cis* regions of *podxl* gene promoter in HGEC. Formaldehyde-crosslinked chromatin fragments were precipitated with either anti-WT1 antibody, anti-RNA polymerase II or no antibody as mock. Precipitated products were amplified by real-time PCR using primers for *podxl* proximal promoter. As normalizer, a DNA fragment lacking any WT1 site, located in the promoter region of GAPDH gene, was used. (**B**) WT1 binds *cis* regions of *nphs1* gene promoter in HGEC. Formaldehyde-crosslinked chromatin fragments were precipitated with either anti-WT1 antibody, anti-RNA polymerase II or no antibody as mock. Precipitated products were amplified by real-time PCR using primers for *nphs1* promoter. As normalizer, a DNA fragment lacking any WT1 site, located in the promoter region of GAPDH gene, was used. (*p<0.05 vs. HGEC:5 mM, #p<0.05 vs. HGEC:25 mM).

WT1 binding elements have been identified on the promoter of PC gene (*podxl*) [24,25]. ChIP followed by quantitative PCR showed that WT1 binding to *podxl* promoter region in HGEC:25 mM was decreased by >50% compared to HGEC:5 mM (Figure 6A columns corresponding to 5 mM and 25 mM). In agreement with the observed permanent reduction of PC expression, binding of WT1 to the *podxl* promoter was also reduced when HGEC:25 mM which do not express PC were reverted to normal glucose for as long as 18 weeks (HGEC:25 mM-to-5 mM/18w) (Figure 7A). Although in HGEC:25 mM-to-5 mM/18w the binding of WT1 to the *podxl* promoter appeared relatively increased compared to that observed in HGEC:25 mM, the increase was not statistically significant. Additionally in HGEC:25 mM-to-5 mM/18w binding of RNA polymerase to the relative promoter region remained reduced. HGEC exposed to 25 mM glucose for 6 weeks (HGEC:5 mM-to-25 mM/6w) displayed a 2-fold reduction of binding of WT1 to *podxl* promoter (Figure 7A), to an extent similar to that observed in HGEC:5 mM-to-25 mM/18w and to that observed in 25 mM glucose (Figure 7A). We then examined whether reduced WT1 binding to *podxl* promoter could be reversed at early (6 weeks) and late (18 weeks) time points. Even though PC expression at the early time point was restored, the binding of WT1 to *podxl* promoter remained decreased (Figure 5 lane 8 and Figure 7A). At the late time point, a partial but non-significant increase of WT1 binding to *podxl* promoter was observed that was not however associated with restoration of PC expression (Figure 5 lane 9 and Figure 7A).

### WT1 binding to the *nphs1* promoter is not affected by high glucose

In addition to the PC promoter, WT1 binds to the nephrin gene (*nphs1*) promoter and activates nephrin expression in podocytes [26]. ChIP followed by quantitative real-time PCR showed that binding of WT1 to the *nphs1* promoter was significantly reduced when HGEC:25 mM which do not express PC were reverted to normal glucose for as long as 18 weeks (HGEC 25 mM-to-5 mM/18w) (Figure 7B). However, there were no significant differences in WT1 binding to the *nphs1* promoter in HGEC:5 mM (expressing nephrin) or HGEC permanently exposed to high glucose (HGEC:25 mM, not expressing nephrin). These results indicate that downregulation and restoration of cell surface-associated nephrin was not accompanied by altered binding of WT1 to the relevant gene promoter (Figure 7B).

## Discussion

The glomerular podocyte is believed to play a role in the development and progression of albuminuria and glomerulosclerosis associated with diabetes, among other [27,28]. Podocytes, and more particularly dysregulation of their differentiation, amongst other injurious stimuli, are at the centre of the pathogenesis of nephropathy. In this study, we describe the gradual modulation of pivotal characteristics of immortalized human podocytes in response to chronic exposure to high glucose. This conversion could be considered as a dedifferentiation process, since it was accompanied by increased expression of mesenchymal vimentin and reduced expression of specialized epithelial components which are podocytic markers. Our data indicated that glucose-mediated PC downregulation which occurred progressively, preceded downregulation of nephrin, the expression of which was substantially suppressed as early as 4 weeks of culture in high glucose.

Changes of podocyte structure and function have been previously described as epithelial-to-mesenchymal transition (EMT) [29-31] since pro-fibrotic components appeared, concomitant with loss of markers characteristic of epithelial differentiation. However, phenotypic changes of podocytes observed *in vitro* or *in vivo* may not necessarily represent EMT-like changes [18]. Podocytes are cells embryonically derived from the metanephric mesenchyme and express epithelial markers (e.g. ZO-1, cytokeratin). Following exposure to TGF-β1, epithelial markers of podocytes were reported to be increased, concomitant with increased tight junction formation [32]. In contrast, in EMT tight junctions are reduced. The phenotypic changes observed in our *in vitro* model more closely resemble a process of partial dedifferentiation. Vimentin, a component of intermediate filaments is expressed in differentiated podocytes but its expression becomes upregulated in podocytes lacking their specific markers, for example in nephrotic glomeruli, in the puromycin aminonucleoside model in rat [22]. Hence enhanced vimentin expression in podocytes following chronic exposure to high glucose could represent a marker of dedifferentiation. Partial podocyte dedifferentiation induced by high glucose could be further supported by the observed loss of PC, nephrin and CALLA, concomitant with upregulation of mesenchymal vimentin.

Our findings are consistent with the reported effects of TGF-β1 and Ang-II resulting in podocyte dedifferentiation and apoptosis under normal and high glucose conditions [7,33-35]. Additionally, our study demonstrates for the first time that dysregulation of the normal podocytic characteristics is an event differentially affecting the expression of function-specific podocytic markers: downregulation of the epithelial marker CD10/CALLA and PC first occurred progressively, and were followed by stably downregulated nephrin at later time intervals.

Nephrin and CD2AP are pivotal for slit diaphragm permselective properties [29,36], and their loss has been linked to podocytic dysregulation and loss of the differentiated podocytic phenotype [37]. The punctate pattern of expression of nephrin and CD2AP which was observed in our *in vitro* system of podocytes (Figures 2, 4) could represent the *in vitro* equivalent of foot-like process formation [11]. Accordingly, glucose-induced, decreased punctate staining in these cells [11] possibly indicated the existence of fewer foot-like processes, resembling foot effacement *in vivo*. Downregulation of the podocyte marker nephrin concomitant with upregulation of the mesenchymal marker vimentin occurred following 4 weeks of exposure to high glucose. Glucose-mediated downregulation of PC expression started as early as two weeks following exposure to high glucose, and gradually reached maximal suppression within 18 weeks. Hence, glucose-induced loss of the differentiated characteristics was complete by 18 weeks. In other studies, in human lung adenocarcinoma, PC downregulation appeared to be intimately associated with upregulation of vimentin and E-cadherin, both involved in mesenchymal transition [38]. We herein report for the first time that in *in vitro* cultured podocytes, PC downregulation was reversible only when the cells still expressed this component in substantial, albeit decreased amounts. At later time intervals, the observed maximal loss of PC became permanent. In agreement with our observations, in an *in vivo* mouse model of podocyte injury, the decrease in nephrin and synaptopodin reflected early downregulation of these proteins in injured but still functioning podocytes, but PC expression was substantially downregulated only in severely injured or sclerotic podocytes [39].

A novel finding of the present study was the observation that restoration of PC expression was not WT1-mediated, since this process was not accompanied by restoration of WT1 binding to the PC gene promoter region. Earlier *in vivo* and *in vitro* studies reported that increased levels of expression and activity of WT1 were associated with increased levels of PC expression in podocytes [25,40]. However, it has been proposed that WT1 alone does not suffice to upregulate PC expression [12,41]. Our observations suggest that WT1 is implicated in establishing basal PC levels and maintaining PC expression in differentiating and differentiated podocytes [25]; nevertheless according to our data WT1 was not directly involved with upregulation of previously reduced PC expression. Moreover, at the late time interval, when PC downregulation had become permanent, a minor (but non-significant) increase of WT1 binding at the relative response element was observed which was not adequate for transcriptional activation. Additional transcription factors could be apparently involved in re-starting the partially or permanently suppressed expression of PC in the presence of high glucose. In this instance, WT1 may function in a manner similar to that used for regulation of the expression of the nephrin gene, since downregulation and re-expression of cell surface-associated nephrin were not accompamied by altered binding of WT1 to the nephrin gene promoter. Interestingly, it has been suggested that the Sp1 zinc finger protein can support transcriptional regulation of either nephrin or PC independently of WT1 [41,42]. Another transcription factor implicated in upregulation of nephrin [43] and PC expression (unpublished data) is the vitamin D receptor (VDR), strongly indicating that enhancement of nephrin and PC expression may be WT1 independent.

## Conclusions

Chronic exposure to high glucose induced a phenotypic conversion of cultured podocytes resembling dedifferentiation. This dedifferentiation process was gradual and progressive, first started with loss of the differentiation markers CD10/CALLA and PC, and was followed by enhanced vimentin and markedly reduced nephrin expression. Reversible upregulation of vimentin expression was associated with restoration of normal nephrin expression. However, PC downregulation was irreversible when maximal loss of PC had been established. These observations indicate that attenuation of PC expression was mainly glucose dependent and persisted in HGEC possessing podocytic characteristics. Dumpening of PC expression could be thus considered a reliable marker of podocytic injury and partial dedifferentiation. Therefore, rescuing PC expression may be pivotal in hyperglycemic conditions such as diabetic nephropathy. However this needs to be verified by *in vivo* studies. Finally, our data suggested that maintenance of the previously established, differentiated podocytic phenotype does not necessarily involve WT1, which however is crucial for the process of differentiation of podocytic presursors to podocytes. Hence, investigating the role of other transcription factors in preserving and restoring structural and functional integrity of the podocytes is of paramount importance.

## Methods

### Cell line and culture conditions in different glucose concentrations

Immortalized T-SV40 HGEC were cultured in the presence of 5 or 25 mM glucose as previously described [11,12]. Cells were released from their culture flasks for passaging by treatment with 0.05% trypsin/0.03% EDTA when they reached 80% confluency. Glucose concentration in the culture medium was adjusted as previously described [12]. Briefly, culturing of immortalized T-SV40 HGEC in the presence of 5 mM glucose was followed by a growing period in the presence of 25 mM with serial passages. During this period the culture medium was changed every 48 hours, with fresh medium containing 25 mM glucose. The duration of this period was up to 18 weeks (HGEC:5-to-25 mM/18w) and a batch of cells was stored in liquid nitrogen once every 2 weeks for the entire period. At the end of the incubation period, the cells were released by trypsin-EDTA from their culture flasks and subsequently used for experiments. Moreover, cell cultures that completed a growing period of 6- or 18- weeks were subsequently plated in tissue culture flasks and allowed to grow in the presence of 5 mM glucose for 4 more weeks (HGEC:25 mM/6w-to-5 mM/4w, HGEC:25 mM/18w-to-5 mM/4w). During this period the culture medium was changed every 48 hours, with fresh medium containing 5 mM glucose. At the end of culture period, cells were released from their flask and used for the relevant experiment.

### Western blotting

Cultured cells were released by trypsin treatment. For total PC and vimentin expression cells were lysed in modified buffer as described previously [11,12]. For total nephrin expression cells were lysed in RIPA buffer. For α3 β1 protein expression cells were lysed in a buffer containing 1% Triton X-100, 1 mM CaCl_2_ as described previously [44]. Protein determination was performed by the Bradford assay (Pierce). For Western blot analysis, 50 μg-100 μg protein from cell lysates were run on 7.5% SDS-PAGE. Proteins were then transferred to Hybond-ECL nitrocellulose membrane (Amersham) for immunoblotting according to previously described procedures [12].

### Flow cytometry (FACS) analysis

Cells were cultured as described, released from their dishes by trypsin treatment, washed with PBS and resuspended in FACS buffer (2% non-heat inactivated FCS in PBS). Cells were incubated with the following antibodies overnight at 4°C; goat anti-nephrin (C-17, Santa Cruz Biotechnology, 1:50), mouse anti-pclp (3D3, Santa Cruz Biotechnology, 1:50) and mouse anti-CD10 (cd-calla, Santa Cruz Biotechnology, 1 μg/10^6^ cells). After washes with FACS buffer cells were incubated with the appropriate Alexa Fluor 488-conjugated secondary antibodies (Invitrogen, 1:1000) and fixed with 1% formaldehyde in PBS. Analysis was performed using Cell Quest Software on a FACScan (Becton Dickinson). To omit debris and cell clumps, gating was performed and 10^4^ gated events were counted. The number of positive cells was calculated in the histogram section selected by the M1 marker in order to subtract the fluorescence of negative control (cells incubated only with the secondary antibody).

### Immunofluorescence

Immunofluorescence studies were performed as described previously [45]. Briefly, coverslip-attached HGEC were fixed in 3.6% paraformaldehyde containing 2% D-sucrose for 15 minutes at room temperature and washed with PBS. For localization of nephrin and CD2AP, cells were permeabilized with HEPES/Triton X-100 buffer (20 mM HEPES, 300 mM D-sucrose, 50 mM NaCl, 3 mM MgCl_2_, 0.5% Triton X-100) for 5 minutes at 4°C; for localization of PC, cells were made permeable with a solution of 0.5% Triton X-100 for 15 minutes at 4°C. Cells were then incubated overnight at 4°C with the following primary antibodies; goat anti-nephrin (C-17, Santa Cruz Biotechnology, 1:20), mouse anti-CD2AP (B-4, Santa Cruz Biotechnology, 1:200), mouse anti-PC (3D3, Santa Cruz Biotechnology, 1:15). After rinsing, slides were incubated with appropriate Alexa Fluor 488-conjugated secondary antibodies (Invitrogen, 1:100) for 1 hour at room temperature, mounted with Vectashield mounting medium and examined. For double staining for CD2AP and actin cells were permeabilized with HEPES/Triton X-100 buffer (20 mM HEPES, 300 mM D-sucrose, 50 mM NaCl, 3 mM MgCl_2_, 0.1% Triton X-100) for 5 minutes at room temperature. Cells were then incubated overnight at 4°C with Texas Red phalloidin (1:100) and mouse anti-CD2AP (1:200) in PBS, 1% calf serum. After rinsing, slides were incubated with the appropriate Alexa Fluor 488-conjugated secondary antibody for CD2AP.

### Chromatin immunoprecipitation assays

Chromatin immunoprecipitation assays were performed with the use of EZ ChIP Chromatin Immunoprecipitation Kit (Millipore) with slight modifications of the supplier’s protocol. HGEC were grown on 10 cm plates until 85% confluency. Proteins were cross-linked to DNA by incubating the cells with 1% formaldehyde in culture medium for 20 minutes at room temperature. Cross-linking was stopped by adding 0.125 M glycine for 5 minutes at room temperature. Cells were collected in PBS containing protease inhibitors cocktail II and centrifuged for 5 minutes at 2000 g at 4°C. Cell pellets were dissolved in 150 mM NaCl, 50 mM Tris pH 8.0, 5 mM EDTA, 0.5% NP-40 and 1% Triton X-100. Nuclei were collected by centrifugation at 12000 g for 5 minutes at 4°C and were suspended in sonication buffer containing 50 mM HEPES, 140 mM NaCl, 1 mM EDTA, 1% Triton X-100, 0.1% sodium deoxycholate, 0.1% SDS and protease inhibitor cocktail II. Aliquots of 350 μl were sonicated in a cold ethanol bath (4 × 5 minutes at 21% intensity with on/off cycles of 30 seconds) with a Sonics Vibra-Cell VCX 750 (Sonics & Materials, Inc.) to an average length of 500 bp and centrifuged at 15000 g for 15 minutes at 4°C. Aliquots of supernatant (100 μl) were incubated overnight at 4°C with 1.2 μg of rabbit anti-WT1 antibody (C19, Santa Cruz Biotechnology), 1.0 μg anti-RNA polymerase II (CTD4H8) or in the absence of antibody. 1% of non-immunoprecipitated chromatin was saved as input sample. After dilution in ChIP dilution buffer, immune complexes were collected by adsorption to protein G-coupled agarose beads for 2 h at 4°C. After stringent washing protein/DNA complexes were eluted from the beads with incubation in 1% SDS, 100 mM NaHCO_3_ for 15 minutes at room temperature. Cross-links between proteins and DNA were reversed by addition of 200 mM NaCl and overnight incubation at 65°C. Following degradation of RNA and proteins, DNA was purified using spin columns. Quantitative amplification of precipitated DNA fragments was performed on a Stratagene Mx3000P system using SYBR Green in triplicate. As normalizer, a DNA fragment lacking any WT1 site was used, located in the promoter region of GAPDH gene. The following primer pairs were used; PC promoter: 5′-TTAATAGATTGGCACAGTTAGG–3′, 5′– GAGAGAAGTTTGGAGAAATACC–3′; *nphs1* promoter: 5′-CGCCCAGTCTCTTTATCTTTC–3′, 5′–GACAAGGAGCAGGAGTGAG– 3′; GAPDH promoter: 5′–TACTAGCGGTTTTACGGGCG–3′, 5′–CGAACAGGAGGAGCAGAGAGCGA-3′. Fold-change in gene promoter site occupancy was calculated as described elsewhere [46].

### Statistical analysis

Results are expressed as means ± SD. Mean values were derived from experiments performed at least three times. Single-factor ANOVA was used to evaluate the results of Western blotting, FACS assays, and ChIP assays. Additionally, post hoc testing using the Newman-Keuls test was used to compare the differences between the selected pairs of means. In all instances, *p<*0.05 was considered statistically significant.

## Abbreviations

PC: Podocalyxin; HGEC: Human Glomerular Epithelial Cells; WT1: Wilms’ Tumor 1; EMT: Epithelial-mesenchymal-transition; SD: Slit diaphragm.

## Competing interests

The authors declared that they have no competing interests.

## Authors’ contributions

NT carried out all of the experimental work and helped drafting the manuscript, GD analysed the data, MS and EK assisted with the immunofluorescence studies, ET and GD participated in the design and coordination of the study, GD drafted the manuscript. All authors have read and approved the final manuscript.

## Supplementary Material

Additional file 1: Figure S1 CD2AP and F-actin colocalization in podocytes. Confocal microscopic analysis for the distribution of CD2AP and F-actin in HGEC. Cells were fixed and stained with Texas Red Phalloidin (red) and anti-CD2AP antibody. Click here for file

Additional file 2: Figure S2 α3 β1-integrin expression in podocytes. (A) Western blot analysis for α3 β1-integrin expression in HGEC. Blots were reprobed with anti-tubulin antibody, to verify protein loads, against which the data were normalized. (B) Results were expressed as the mean ± SD of three independent experiments (*p<0.05 vs. HGEC:5 mM, #p<0.05 vs. HGEC:25 mM). Click here for file
