# Supplementary figures and images for: Glucose-induced gradual phenotypic modulation of cultured human glomerular epithelial cells may be independent of Wilms’ tumor 1 (WT1)
Source: BMC Cell Biol. 2013 Jun 14;14:28. doi: 10.1186/1471-2121-14-28 (PMC3686613; doi:10.1186/1471-2121-14-28)

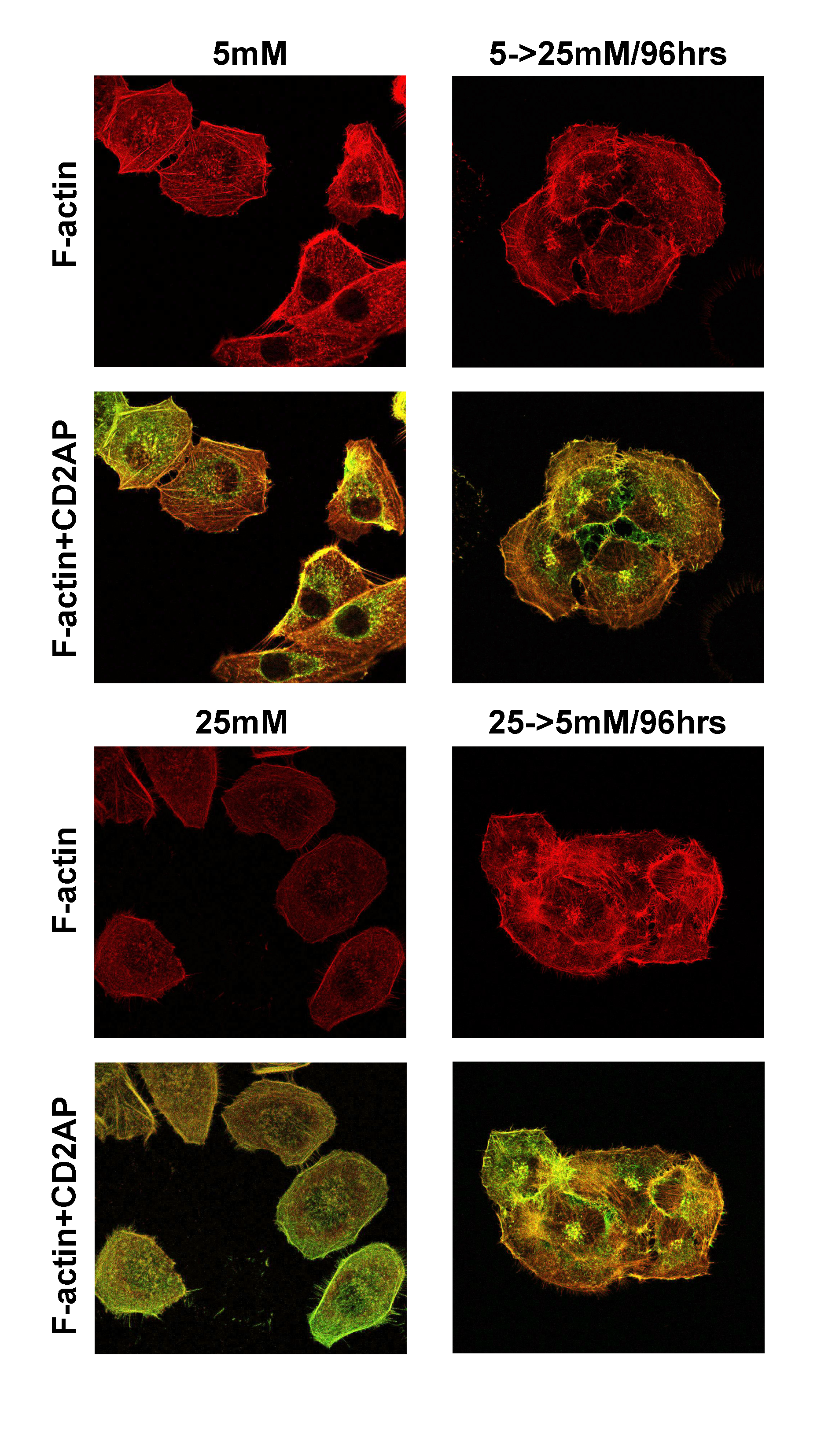

Supplement: Additional file 1: Figure S1 — CD2AP and F-actin colocalization in podocytes. Confocal microscopic analysis for the distribution of CD2AP and F-actin in HGEC. Cells were fixed and stained with Texas Red Phalloidin (red) and anti-CD2AP antibody. [file 1471-2121-14-28-S1.tiff]

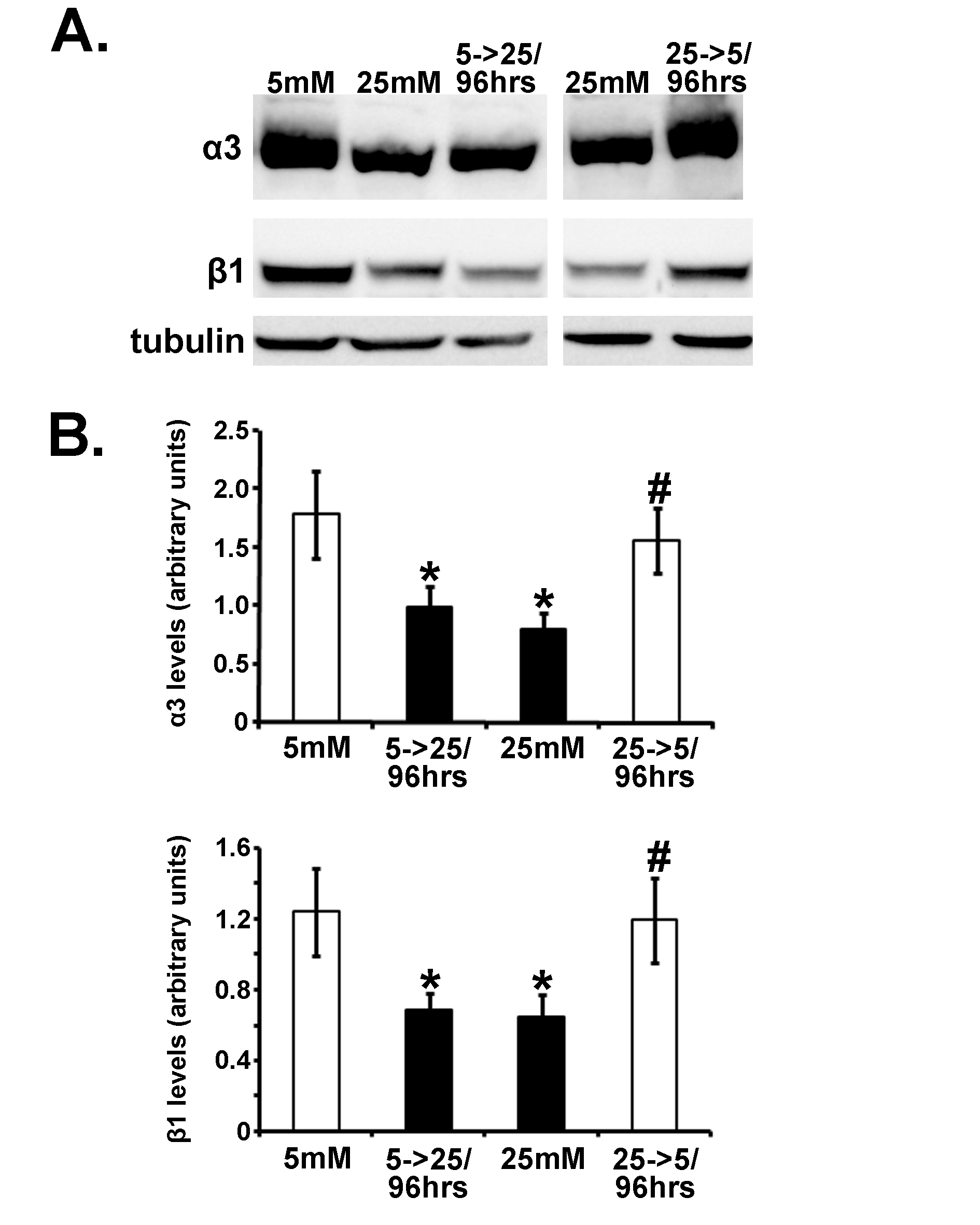

Supplement: Additional file 2: Figure S2 — α3 β1-integrin expression in podocytes. (A) Western blot analysis for α3 β1-integrin expression in HGEC. Blots were reprobed with anti-tubulin antibody, to verify protein loads, against which the data were normalized. (B) Results were expressed as the mean ± SD of three independent experiments (*p<0.05 vs. HGEC:5 mM, #p<0.05 vs. HGEC:25 mM). [file 1471-2121-14-28-S2.tiff]
